# Prophylactic Anticoagulation and Thrombosis in Hospitalized Patients with Clinically Stable COVID-19 at Admission: From the Practice-Based Observational Study

**DOI:** 10.3400/avd.oa.23-00031

**Published:** 2023-11-28

**Authors:** Yugo Yamashita, Sen Yachi, Makoto Takeyama, Yuji Nishimoto, Ichizo Tsujino, Junichi Nakamura, Naoto Yamamoto, Hiroko Nakata, Satoshi Ikeda, Michihisa Umetsu, Shizu Aikawa, Hiroya Hayashi, Hirono Satokawa, Yoshinori Okuno, Eriko Iwata, Yoshito Ogihara, Nobutaka Ikeda, Akane Kondo, Takehisa Iwai, Norikazu Yamada, Tomohiro Ogawa, Takao Kobayashi, Makoto Mo, on behalf of the Clot-COVID Study Investigators

**Affiliations:** 1 Department of Cardiovascular Medicine, Kyoto University Hospital, Kyoto, Kyoto, Japan; 2 Japan Community Health Care Organization Tokyo Shinjuku Medical Center, Tokyo, Japan; 3 Hyogo Prefectural Amagasaki General Medical Center, Amagasaki, Hyogo, Japan; 4 Hokkaido University Hospital, Sapporo, Hokkaido, Japan; 5 Hamamatsu Medical Center, Hamamatsu, Shizuoka, Japan; 6 Yokosuka General Hospital Uwamachi, Yokosuka, Kanagawa, Japan; 7 Nagasaki University Graduate School of Biomedical Sciences, Nagasaki, Nagasaki, Japan; 8 Tohoku University Hospital, Sendai, Miyagi, Japan; 9 Tsukuba Medical Center Hospital, Tsukuba, Ibaraki, Japan; 10 Osaka Metropolitan University Graduate School of Medicine, Suita, Osaka, Japan; 11 Fukushima Medical University, School of Medicine, Fukushima, Fukushima, Japan; 12 Nankai Medical Center Japan Community Health Care Organization, Saiki, Oita, Japan; 13 Mie University Hospital, Tsu, Mie, Japan; 14 Toho University Ohashi Medical Center, Tokyo, Japan; 15 Shikoku Medical Center for Children and Adults, Zentsuji, Kagawa, Japan; 16 Tsukuba Vascular Center, Moriya, Ibaraki, Japan; 17 Kuwana City Medical Center, Kuwana, Mie, Japan; 18 Fukushima Daiich Hospital, Fukushima, Fukushima, Japan; 19 Yokohama Minami Kyosai Hospital, Yokohama, Kanagawa, Japan

**Keywords:** COVID-19, thrombosis, stable, mild, anticoagulation

## Abstract

**Objectives:** The potential benefit of routine prophylactic anticoagulation for all hospitalized patients with clinically stable coronavirus disease 2019 (COVID-19) is still controversial.

**Method:** The CLOT-COVID Study was a multicenter observational study enrolling 2894 consecutive hospitalized patients with COVID-19. The current study population consisted of 1738 hospitalized patients with mild COVID-19 at admission not requiring oxygen administration, who were divided into 2 groups: patients with prophylactic anticoagulation (n = 326) and those without (n = 1412).

**Results:** Patients with prophylactic anticoagulation had more severe status of the worst severity of COVID-19 during hospitalization compared with those without (mild: 38% versus 82%, moderate: 55% versus 17%, and severe or death at discharge: 6.4% versus 0.7%, P <0.001). During hospitalization, 8 patients (0.5%) developed thrombosis, and the incidences of thrombosis were numerically higher in patients with more severe status of worst severity of COVID-19 during hospitalization (mild: 0.2%, moderate: 1.2%, and severe or death at discharge: 3.2%).

**Conclusions:** Among hospitalized patients with clinically stable COVID-19 at admission, patients who did not worsen in COVID-19 severity after admission rarely developed thrombosis, although patients with worsening of COVID-19 severity after admission more often received prophylactic anticoagulation and might have a higher risk of thrombosis.

## Introduction

The coronavirus disease 2019 (COVID-19) has become a huge threat all over the world,^1^^,^^2)^ which has been reported to cause cardiovascular complications including thrombosis.^3^^,^^4)^ Based on the potential benefit of prophylactic anticoagulation for prevention of thrombosis and worsening of disease severity, several international statements recommend prophylactic anticoagulation for all hospitalized patients with COVID-19.^5^^,^^6)^ However, the risk of thrombosis in patients with COVID-19 has been reported to vary widely according to patient characteristics including disease severity of COVID-19, and optimal prophylactic anticoagulation strategies could be still controversial.^7^^,^^8)^

Because patients with severe status of COVID-19 have been reported to be at a higher risk of thrombosis, prophylactic anticoagulation for patients with clinically unstable COVID-19 could be useful to improve clinical outcomes.^9^^,^^10)^ Previous randomized clinical trials have evaluated the potential benefit of prophylactic anticoagulation among hospitalized patients with more severe status of COVID-19, including those requiring oxygen administration and mechanical ventilation.^11^^–^^14)^ However, the potential benefit of prophylactic anticoagulation for all hospitalized patients with clinically stable COVID-19 including those who do not require oxygen administration might be a matter of active debate. Actually, a recent study has failed to show a potential benefit of antithrombotic therapy for symptomatic clinically stable outpatients with COVID-19.^15)^ Because the majority of patients with COVID-19 are currently on clinically stable status, the optimal prophylactic anticoagulation strategy for those patients is becoming more clinically relevant, although there has been still limited data on the issue. Thus, the current study aimed to overview the current status of prophylactic anticoagulation and thrombosis in hospitalized patients with clinically stable COVID-19, using a large observational database of patients with COVID-19 in Japan.

## Materials and Methods

### Study population

The CLOT-COVID Study (Thrombosis and Anticoagulation Therapy in Patients with COVID-19 in Japan Study: UMIN000045800) was a multicenter retrospective cohort study enrolling 2894 consecutive hospitalized patients with COVID-19 at 16 centers in Japan between April 2021 and September 2021. The details of the study were reported previously.^16^^,^^17)^ The relevant review boards or ethics committees in all participating centers (Supplementary Appendix 1; all supplementary files are available online) approved the research protocol.

**Figure 1:**
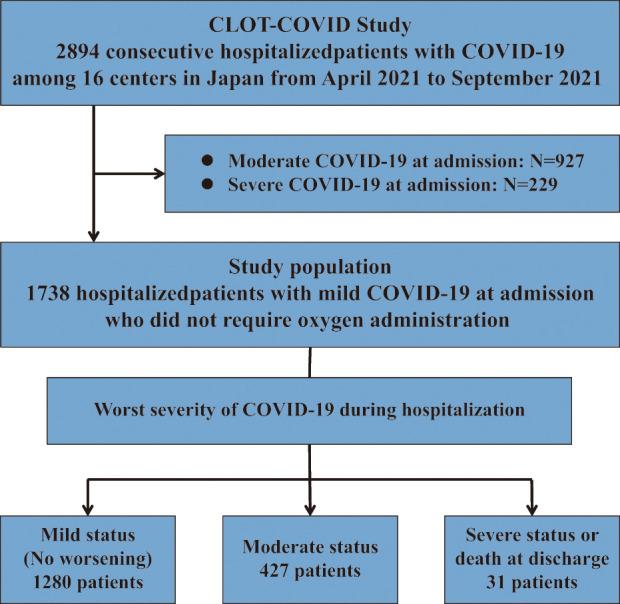
Fig. 1 Study flow chart. Patients with mild COVID-19 were defined as those who did not require oxygen, patients with moderate COVID-19 were defined as those who required oxygen, and patients with severe COVID-19 were defined as those who required mechanical ventilation or ECMO. COVID-19: coronavirus disease 2019; ECMO: extracorporeal membrane oxygenation

In the current study, after excluding 927 patients with moderate COVID-19 at admission who required oxygen administration and 229 patients with severe COVID-19 at admission who required mechanical ventilation or extracorporeal membrane oxygenation (ECMO),^10^^,^^18)^ the current study population consisted of 1738 hospitalized patients with mild COVID-19 at admission who did not require oxygen administration (****Fig**. **1****). The current study population was divided into 2 groups: patients with prophylactic anticoagulation during hospitalization and those without, and we compared patient characteristics and clinical outcomes during hospitalization between the 2 groups.

### Definitions for patient characteristics

Prophylactic anticoagulation was defined as the usage of anticoagulants during the hospitalization except for their usage for the treatment of thrombosis, including unfractionated heparin, low-molecular-weight heparin, direct oral anticoagulants, and warfarin. Worst severity of COVID-19 during hospitalization was classified into 3 groups: mild status (no worsening after admission), moderate status who required oxygen administration, and severe status who required mechanical ventilation or ECMO, or death at discharge. The detailed definitions of other patient characteristics are described in the Supplementary Appendix 2.

### Clinical endpoints

The primary endpoint was thrombosis during the hospitalization, which included venous thromboembolism (VTE), ischemic stroke, myocardial infarction, systemic arterial thromboembolism, and other systemic thrombosis. VTE was defined as pulmonary embolism and/or deep vein thrombosis that was objectively confirmed by imaging examinations or by autopsy. Ischemic stroke was defined as stroke either requiring or prolonging the hospitalization with symptoms lasting more than 24 hours. Myocardial infarction was defined in accordance with the universal myocardial infarction guidelines.^19)^

The secondary endpoints were major bleeding and all-cause death during hospitalization. Major bleeding was diagnosed as International Society of Thrombosis and Hemostasis (ISTH) major bleeding.^20)^

### Statistical analysis

Categorical variables were presented as numbers and percentages, which were compared with the chi-square test when appropriate; otherwise, a Fisher’s exact test was used. Continuous variables were presented as mean and standard deviation or median and interquartile range based on their distributions, which were compared using the Student’s t-test or the Wilcoxon rank-sum test. The clinical endpoints were presented as numbers of events and percentages with the 95% confidence intervals (CIs). Furthermore, we evaluated clinical endpoints by stratified analysis according to the worst severity of COVID-19 during hospitalization. All statistical analyses were performed using JMP version 14.0 software (SAS Institute Inc., Cary, NC, USA). All reported P-values were 2-tailed, and P-values less than 0.05 were considered significant statistically.

## Results

### Patient characteristics

Among 1738 hospitalized patients with mild COVID-19 at admission, 326 patients (19%) received prophylactic anticoagulation during hospitalization and 1412 patients (81%) did not. Patients with prophylactic anticoagulation during hospitalization were older (56.7 years versus 47.0 years, P <0.001), more often men (70% versus 60%, P <0.001), and had a higher body weight and body mass index (71.2 kg versus 66.1 kg, P <0.001 and 26.1 kg/m^2^ versus 24.4 kg/m^2^, P <0.001) than those without (**Table 1**). The median D-dimer level at admission was higher in patients with prophylactic anticoagulation than that in those without (0.8 μg/mL versus 0.5 μg/mL, P <0.001). Patients with prophylactic anticoagulation during hospitalization more often had several comorbidities including hypertension, diabetes mellitus, heart disease, and active cancer.

**Table:** Table 1 Patient characteristics and management strategies during hospitalization

	Mild COVID-19 at admission (n = 1738)	Prophylactic anticoagulation during hospitalization (n = 326)	No prophylactic anticoagulation during hospitalization (n = 1412)	P-value
Baseline characteristics				
Age (years)	48.8 ± 18.9	56.7 ± 16.3	47.0 ± 19.0	<0.001
Men	1075 (62%)	228 (70%)	847 (60%)	<0.001
Body weight (kg)	67.1 ± 19.0	71.2 ± 17.8	66.1 ± 19.1	<0.001
Body mass index (kg/m^2^)	24.7 ± 5.3	26.1 ± 5.5	24.4 ± 5.2	<0.001
Body mass index >30 kg/m^2^	247 (14%)	66 (20%)	181 (13%)	<0.001
D-dimer level at admission (μg/mL) (n = 1643)	0.6 (0.5-0.9)	0.8 (0.5–1.3)	0.5 (0.5-0.8)	<0.001
Comorbidities				
Hypertension	414 (24%)	124 (38%)	290 (21%)	<0.001
Diabetes mellitus	283 (16%)	75 (23%)	208 (15%)	<0.001
Heart disease	126 (7.3%)	48 (15%)	78 (5.5%)	<0.001
Respiratory disease	158 (9.1%)	31 (9.5%)	127 (9.0%)	0.77
Active cancer	33 (1.9%)	11 (3.4%)	22 (1.6%)	0.03
History of major bleeding	12 (0.7%)	3 (0.9%)	9 (0.6%)	0.58
History of VTE	8 (0.5%)	3 (0.9%)	5 (0.4%)	0.17
Worst severity of COVID-19 during hospitalization				
Mild (no worsening after admission)	1280 (74%)	125 (38%)	1155 (82%)	<0.001
Moderate (need oxygen)	427 (25%)	180 (55%)	247 (17%)	
Severe (need mechanical ventilation/ECMO) or death at discharge	31 (1.8%)	21 (6.4%)	10 (0.7%)	
Prophylactic anticoagulation during hospitalization				
Unfractionated heparin of a prophylactic dose	–	197 (60%)	–	–
Unfractionated heparin of a therapeutic dose	–	11 (3.4%)	–	–
Low-molecular-weight heparin of a prophylactic dose	–	71 (22%)	–	–
Low-molecular-weight heparin of a therapeutic dose	–	0 (0%)	–	–
Direct oral anticoagulants	–	31 (9.5%)	–	–
Warfarin	–	10 (3.1%)	–	–
Others	–	6 (1.8%)	–	–
Imaging examinations during hospitalization				
Contrast-enhanced CT examination	22 (1.3%)	11 (3.4%)	11 (0.8%)	<0.001
Ultrasound examination of the lower extremities	7 (0.4%)	4 (1.2%)	3 (0.2%)	0.009

Categorical variables are presented as numbers and percentages, and continuous variables are presented as mean and standard deviation or median and interquartile range based on their distributions. Categorical variables were compared using the chi-squared test when appropriate; otherwise, the Fisher’s exact test was used. Continuous variables were compared using the Student’s t test or Wilcoxon rank-sum test based on distribution.

Prophylactic anticoagulation was evaluated by the usage of any anticoagulants during the hospitalization except for their usage for the treatment of thrombosis. Unfractionated heparin of a therapeutic dose was defined as the administration of unfractionated heparin targeting a therapeutic range referencing the APTT. Unfractionated heparin of a prophylactic dose was defined as the administration of unfractionated heparin of a fixed dose without referencing the APTT.

VTE, venous thromboembolism; COVID-19, coronavirus disease 2019; ECMO, extracorporeal membrane oxygenation; CT, computed tomography; APTT, activated partial thromboplastin time.

Patients with prophylactic anticoagulation during hospitalization had more severe status of the worst severity of COVID-19 during hospitalization compared with those without (mild: 38% versus 82%, moderate: 55% versus 17%, and severe or death at discharge: 6.4% versus 0.7%, P <0.001) (**Table 1**). Most of prophylactic anticoagulation was conducted with anticoagulants of a prophylactic dose, and 60% and 22% of prophylactic anticoagulation was unfractionated heparin of a prophylactic dose and low-molecular-weight heparin of a prophylactic dose, respectively (**Table 1**).

### Clinical endpoints

During hospitalization, 8 patients (0.5% [95% CI, 0.2%–0.9%]) developed thrombosis, and patients with prophylactic anticoagulation during hospitalization more often developed thrombosis than those without (2.5% [95% CI, 1.2%–4.9%] versus 0.0% [95% CI, 0.0%–0.3%], P <0.001) (**Table 2**). The incidences of thrombosis were numerically higher in patients with more severe status of worst severity of COVID-19 during hospitalization (mild: 0.2%, moderate: 1.2%, and severe or death at discharge: 3.2%) (**Table 3**). Among 1280 patients with mild status of worst severity of COVID-19 during hospitalization (no worsening after admission), 2 patients developed thrombosis whose D-dimer levels at admission were 4.6 μg/mL and 15.0 μg/mL, respectively (**Table 4**).

**Table:** Table 2 Clinical outcomes during hospitalization

	Mild COVID-19 at admission (n = 1738)	Prophylactic anticoagulation during hospitalization (n = 326)	No prophylactic anticoagulation during hospitalization (n = 1412)	P-value
Thrombosis	8 (0.5% [0.2%–0.9%])	8 (2.5% [1.2%–4.9%])	0 (0.0% [0.0%–0.3%])	<0.001
VTE	3 (0.2% [0.0%–0.5%])	3 (0.9% [0.2%–2.8%])	–	–
Arterial thrombotic events	2 (0.1% [0.0%–0.4%])	2 (0.6% [0.0%–2.4%])	–	–
Ischemic stroke	2/2 (100%)	2/2 (100%)	–	–
Myocardial infarction	0/2 (0%)	0/2 (0%)	–	–
Systemic arterial thromboembolism	0/2 (0%)	0/2 (0%)	–	–
Other thrombosis	3 (0.2% [0.0%–0.5%])	3 (0.9% [0.2%–2.8%])	–	–
Major bleeding	8 (0.5% [0.2%–0.9%])	4 (1.2% [0.4%–3.2%])	4 (0.3% [0.1%–0.8%])	0.02
All-cause death	25 (1.4% [1.0%–2.1%])	16 (4.9% [3.0%–7.9%])	9 (0.6% [0.3%–1.2%])	<0.001

The clinical outcomes are presented as numbers of events and percentages with 95% CIs, which were compared using the chi-squared test when appropriate; otherwise, the Fisher’s exact test was used.

COVID-19: coronavirus disease 2019; VTE: venous thromboembolism; CI: confidence interval

**Table:** Table 3 Clinical outcomes during hospitalization stratified by worst severity of COVID-19 during hospitalization

	Mild COVID-19 at admission	Prophylactic anticoagulation during hospitalization	No prophylactic anticoagulation during hospitalization
Mild status (n = 1280)			
Thrombosis	2/1280 (0.2%)	2/125 (1.6%)	0/1155 (0.0%)
Major bleeding	3/1280 (0.2%)	0/125 (0.0%)	3/1155 (0.3%)
Moderate status (n = 427)			
Thrombosis	5/427 (1.2%)	5/180 (2.8%)	0/247 (0.0%)
Major bleeding	3/427 (0.7%)	2/180 (1.1%)	1/247 (0.4%)
Severe status or death at discharge (n = 31)			
Thrombosis	1/31 (3.2%)	1/21 (4.8%)	0/10 (0.0%)
Major bleeding	2/31 (6.5%)	2/21 (9.5%)	0/10 (0.0%)

The clinical outcomes are presented as numbers of events and percentages.

COVID-19: coronavirus disease 2019

**Table:** Table 4 Detailed individual cases who developed thrombosis during hospitalization

	Patient 1	Patient 2	Patient 3	Patient 4	Patient 5	Patient 6	Patient 7	Patient 8
Worst severity of COVID-19 during hospitalization	Mild status	Mild status	Moderate status	Moderate status	Moderate status	Moderate status	Moderate status	Death at discharge
Age (years)	59	61	44	57	62	67	70	75
Sex	Men	Men	Men	Men	Men	Men	Men	Men
Body weight (kg)	52.6	66.0	90.0	90.0	75.0	55.1	69.0	60.0
Body mass index (kg/m^2^)	19.8	19.7	28.7	30.4	25.1	19.4	24.2	24.7
D-dimer level at admission (μg/mL)	4.6	15.0	2.9	1.9	1.2	2.5	1.3	2.3
Comorbidities	Hypertension	None	None	Hypertension	None	Hypertension, diabetes mellitus, heart disease	Hypertension, diabetes mellitus	Heart disease
Prophylactic anticoagulation during hospitalization	Yes	Yes	Yes	Yes	Yes	Yes	Yes	Yes
Types of thrombosis	Portal vein thrombus	Ischemic stroke	VTE (PE + DVT)	Ischemic stroke	VTE (DVT)	Ventricular thrombus	VTE (PE)	Systemic arterial and venous thrombosis

COVID-19: coronavirus disease 2019; VTE: venous thromboembolism; PE: pulmonary embolism; DVT: deep vein thrombosis

During hospitalization, 8 patients (0.5% [95% CI, 0.2%–0.9%]) developed major bleeding and 25 patients (1.4% [95% CI, 1.0%–2.1%]) died including 17 deaths (68%) due to COVID-19 related respiratory failure (**Table 2**). Patients with prophylactic anticoagulation during hospitalization more often developed major bleeding than those without (1.2% [95% CI, 0.4%–3.2%] versus 0.3% [95% CI, 0.1%–0.8%], P = 0.02).

## Discussion

The main findings of the current study are as follows: 1) Patients who worsened in COVID-19 severity after admission more often received prophylactic anticoagulation during hospitalization, 2) the incidence of thrombosis was numerically higher in patients who worsened in COVID-19 severity after admission than that in those who did not, and 3) a minority of patients without worsening of COVID-19 severity developed thrombosis, who showed relatively high D-dimer levels at admission.

Thrombosis is reported to be one of common complications in patients with COVID-19 including thrombus formation in large vessels as well as in microvasculature, which has been described as COVID-19-associated coagulopathy.^21)^ COVID-19-associated coagulopathy was reported to cause in-situ thrombosis in large and small vessels of lung at the capillary–alveolar interface, which might contribute to the worsening of respiratory failure.^22^^,^^23)^ Thus, there has been thought to be potential benefit of prophylactic anticoagulation for patients with COVID-19. Actually, several observational studies reported that anticoagulation therapy during hospitalization was associated with better clinical outcomes, including improved survival in hospitalized patients with COVID-19.^24^^,^^25)^ Based on these previous reports, several current guidelines recommend prophylactic anticoagulation for all hospitalized patients with COVID-19.^5^^,^^6)^ On the other hand, the threshold for admission to the hospital could widely vary according to each region and country due to a different medical system and resource availability, as well as a different study period. In Japan, the hospitalization for COVID-19 has not been limited to only patients with severe status of COVID-19 and a certain number of patients with clinically stable COVID-19 were admitted to the hospital. The latest Japanese domestic COVID-19 Clinical Practice Guidelines by the Ministry of Health, Labour, and Welfare in Japan has recommended that patients with clinically unstable COVID-19 requiring oxygen administration should be considered for prophylactic anticoagulation, but has not recommended routine prophylactic anticoagulation for patients with clinically stable COVID-19 not requiring oxygen administration. The current study showed only approximately 20% of hospitalized patients with mild COVID-19 at admission received prophylactic anticoagulation during hospitalization, and patients who worsened in COVID-19 severity after admission were more likely to receive prophylactic anticoagulation, which suggested that many clinicians did not frequently conduct prophylactic anticoagulation for patients with clinically stable COVID-19 in daily clinical practice if they kept clinically stable until discharge.

The current study also showed that the risk of thrombosis might become higher in patients who worsened in COVID-19 severity after admission. Considering a higher risk of thrombosis in patients with more severe status of COVID-19,^9^^,^^10)^ initiation of prophylactic anticoagulation for patients with clinically stable COVID-19 at admission could be useful if they worsened in COVID-19 severity after admission. Because the COVID-19 severity could be changed dynamically through the course of treatment even in clinically stable patients at admission,^1^^,^^26)^ clinicians might have to be notified that the risk of thrombosis could be changed according to the status of COVID-19 severity after admission. In line with the current study, a previous study reported that 3.3% of clinically stable outpatients with COVID-19 became clinically unstable during the follow-up period and a few of them died due to COVID-19-related respiratory failure.^15)^ These results might suggest the importance of close medical follow-up even in patients with initially clinically stable COVID-19.

Although several current guidelines recommend prophylactic anticoagulation for all hospitalized patients with COVID-19,^5^^,^^6)^ the necessity of routine prophylactic anticoagulation for all hospitalized patients with clinically stable COVID-19 could be controversial. There have been limited studies evaluating prophylactic anticoagulation specifically for hospitalized patients with clinically stable COVID-19. The recent randomized clinical trial evaluating outpatients with COVID-19 has reported that prophylactic anticoagulation compared with placebo did not reduce the major adverse outcomes in patients with clinically stable COVID-19,^15)^ suggesting no benefit of prophylactic anticoagulation for clinically stable outpatients. The current study showed that a minority of patients without worsening of COVID-19 severity after admission developed thrombosis, which could suggest a quite low risk of thrombosis in patients with clinically stable COVID-19. The current study revealed that among 1280 patients with clinically stable COVID-19 during hospitalization, only 2 patients (0.2%) developed thrombosis. One of clinical features in these patients was relatively high D-dimer levels at admission of 4.6 μg/mL and 15.0 μg/mL. In patients with clinically stable COVID-19, D-dimer levels at admission might be important for risk assessment for development of thrombosis beyond the COVID-19 severity. Although it could be difficult to estimate the effect of prophylactic anticoagulation for these patients, the current results could be useful for clinicians because there was no signal of concerns for the management strategy without prophylactic anticoagulation for these low-risk patients. The current study could challenge the potential benefit of routine prophylactic anticoagulation for all hospitalized patients with clinically stable COVID-19. Considering the risk of bleeding related to prophylactic anticoagulation, further randomized clinical trials should be warranted to investigate the potential benefit and harm of prophylactic anticoagulation for hospitalized patients with clinically stable COVID-19.

### Study limitations

The current study had several limitations. First and most importantly, the current study was an observational study, which could show only association, not causality. Especially, causal relationship between the prophylactic anticoagulation and development of thrombosis was unclear. Overlapping risk factors for thrombosis and COVID-19 severity could be a potential mechanism for the current results. In addition, the therapeutic decision-making including prophylactic anticoagulation was left to the discretion of the attending physicians, which could have a certain influence on clinical outcomes. Second, the absolute number of clinical events was relatively small, although it was derived from a large observational database of patients with COVID-19. We could not conduct the statistical adjustment including the multivariable analyses to take baseline imbalances between the groups into consideration. Third, the current study evaluated only clinical outcomes during hospitalization. Thus, we could not discuss the risk of thrombosis after discharge. Fourth, the vast majority of virus strain in the current study period (from April 2021 to September 2021) was assumed to be delta variant, and the current results could be applied to COVID-19 with the delta variant. The generalizability of the current study for the COVID-19 with other variants should be carried out carefully. Finally, the demographics and practice patterns as well as the clinical outcomes in Japan may differ from those outside Japan. Thus, it should be interpreted with caution whether the current results could be extrapolated to patients in different regions and countries.

## Conclusions

Among hospitalized patients with clinically stable COVID-19 at admission, patients who did not worsen in COVID-19 severity after admission rarely developed thrombosis, although patients with worsening of COVID-19 severity after admission more often received prophylactic anticoagulation and might have a potential higher risk of thrombosis.

## Acknowledgments

The CLOT-COVID study was partially supported by research funding from Fujiwara Memorial Foundation (Kyoto, Japan) and research funding from Foundation Kyoto Health Care Society (Kyoto, Japan).

## Disclosure Statement

All authors have reported that they have no relationships relevant to the contents of this paper to disclose.

## Author Contributions

Study conception: YY

Data collection: all authors

Analysis: YY

Investigation: all authors

Writing: YY

Critical review and revision: all authors

Final approval of the article: all authors

Accountability for all aspects of the work: all authors.

## Supplementary Material

Supplementary Appendix 1: List of participating centers and investigators

Supplementary Appendix 2: Definitions for patient characteristics
