# Supplementary material for: Prophylactic Anticoagulation and Thrombosis in Hospitalized Patients with Clinically Stable COVID-19 at Admission: From the Practice-Based Observational Study
Source: Ann Vasc Dis. 2023 Nov 28;17(1):1–8. doi: 10.3400/avd.oa.23-00031 (PMC11018098; doi:10.3400/avd.oa.23-00031)
Supplement: Supplementary Appendix 1: — List of participating centers and investigators [file avd-17-1-s01.pdf]

Supplementary Appendix 1: List of participating centers and investigators

Kyoto University Hospital: Yugo Yamashita, Yoshinori Okuno (Kyoto University Hospital Ethics Committee; the approval number R3239)

Japan Community Health Care Organization Tokyo Shinjuku Medical Center: Sen Yachi, Makoto Takeyama (Tokyo Shinjuku Medical Center Ethics Committee; the approval number R3-23)

Hyogo Prefectural Amagasaki General Medical Center: Yuji Nishimoto (Amagasaki General Medical Center Ethics Committee; the approval number 3-93)

Hokkaido University Hospital: Ichizo Tsujino, Junichi Nakamura (Hokkaido University Hospital Ethics Committee; the approval number 2021)

Hamamatsu Medical Center: Naoto Yamamoto, Takao Kobayashi (Hamamatsu Medical Center Ethics Committee; the approval number 2021-3-061)

Yokosuka General Hospital Uwamachi: Hiroko Nakata (Yokosuka General Hospital Ethics Committee; the approval number 2021)

Nagasaki University Graduate School: Satoshi Ikeda (Nagasaki University Graduate School Ethics Committee; the approval number 21122007)

Tohoku University Hospital: Michihisa Umetsu (Tohoku University Hospital Ethics Committee; the approval number 2021)

Tsukuba Medical Center Hospital: Shizu Aikawa (Tsukuba Medical Center Hospital Ethics Committee; the approval number 2021-001)

Osaka Metropolitan University Graduate School of Medicine: Hiroya Hayashi (Osaka Metropolitan University Ethics Committee: the approval number 2021-221)

Fukushima Medical University: Hirono Satokawa (Fukushima Medical University Ethics Committee: the approval number 2021-235)

Nankai Medical Center Japan Community Health Care Organization: Eriko Iwata (Nankai Medical Center Ethics Committee: the approval number 2021)

Mie University Hospital: Yoshito Ogihara (Mie University Hospital Ethics Committee: the approval

number H2021-223)

Toho University Ohashi Medical Center: Nobutaka Ikeda (Toho University Ohashi Medical Center Ethics Committee: the approval number H21083)

Shikoku Medical Center for Children and Adults: Akane Kondo (Shikoku Medical Center for Children and Adults Ethics Committee: the approval number R03-31)

Yokohama Minami Kyosai Hospital: Makoto MO (Yokohama Minami Kyosai Hospital Ethics Committee: the approval number 1-21-11-1)
