# Supplementary material for: Prophylactic Anticoagulation and Thrombosis in Hospitalized Patients with Clinically Stable COVID-19 at Admission: From the Practice-Based Observational Study
Source: Ann Vasc Dis. 2023 Nov 28;17(1):1–8. doi: 10.3400/avd.oa.23-00031 (PMC11018098; doi:10.3400/avd.oa.23-00031)
Supplement: Supplementary Appendix 2: — Definitions for patient characteristics [file avd-17-1-s02.pdf]

Supplementary Appendix 2: Definitions for patient characteristics

Hypertension was diagnosed if the peripheral blood pressure was >140/90mmHg or if the patient was taking medication for hypertension. The presence of diabetes was diagnosed using the hemoglobin A1c (HbA1c) [National Glycohemoglobin Standardization Program (NGSP), 6.5%] as the standard or was assumed if the patient was taking medication for the treatment of diabetes. Heart disease was defined as heart disorders such as heart failure, angina pectoris, and a history of myocardial infarction. Heart failure was diagnosed if the patient had a history of a hospitalization for heart failure, the patient had symptoms due to heart failure (New York Heart Association [NYHA] functional class ≥2), or the left ventricular ejection fraction was <40%. Respiratory disease was defined as a persistent lung disorder such as asthma, chronic obstructive pulmonary disease, or restrictive lung disease. Patients with active cancer were defined as those on treatment for cancer, such as chemotherapy or radiotherapy, those scheduled to undergo cancer surgery, those with metastasis to other organs, and/or those with terminal cancer[27]. A history of major bleeding was diagnosed if the patient had a history of International Society of Thrombosis and Hemostasis (ISTH) major bleeding, which consisted of a reduction in the hemoglobin level by at least 2 g/dL, transfusion of at least 2 units of blood, or symptomatic bleeding in a critical area or organ[20]. Unfractionated heparin of a therapeutic dose was defined as the administration of unfractionated heparin targeting a therapeutic range referencing the activated partial thromboplastin time (APTT), and unfractionated heparin of a prophylactic dose was defined as the administration of unfractionated heparin of a fixed dose without a referencing the APTT.
